# Subcutaneous Reaction of Rat Tissues to Nanosilver Coated Gutta-Percha

**DOI:** 10.22037/iej.2017.31

**Published:** 2017

**Authors:** Mohammad Ali Mozayeni, Omid Dianat, Shahin Tahvildari, Maryam Mozayani, Payam Paymanpour

**Affiliations:** a *Department of Endodontics, Dental School, Shahid Beheshti University of Medical Sciences, Tehran, Iran; *; b *Department of orthodontics, Shahid Sadoughi University of Medical Sciences, Yazd, Iran; *; c * University at buffalo School of Dental Medicine, NY, USA*

**Keywords:** Gutta-Percha, Inflammation, Nanosilver Coated Gutta-Percha, Subcutaneous Connective Tissues

## Abstract

**Introduction:**

Gutta-percha (GP), is a neutral and non-toxic material. The aim of this animal study was to compare the biocompatibility of nanosilver coated GP (NS-GP) with conventional GP in subcutaneous tissues in a rat model.

**Methods and Materials:**

Conventional GP and NS-GP were subcutaneously implanted in the backs of 20 male Wistar rats (*n*=10). A control animal was assigned for each trial period. Ten animals were sacrificed after 7 and 30 days and light microscopic evaluation of tissue reaction to NS-GP (*n*=20) and conventional GP (*n*=20) was accomplished. The Mann-Whitney U, Wilcoxon Signed Ranks, Fisher Exact, and McNemar tests were used for statistical analysis of the data.

**Results:**

After 7 days, inflammation was moderate and mild for NS-GP and conventional GP, respectively (*P*<0.001). After 30 days, no inflammation was discernible in conventional GP. However, mild inflammation was reported for NS-GP (*P*<0.001). Regarding inflammatory cell type, there was a significant difference between two experimental groups at both times (*P*<0.001).

**Conclusion:**

Inflammation decreased over time in both groups. Fibrous connective tissue, a representative of healing and control of inflammatory process, surrounded both test materials. NS-GP was biocompatible and might be a reasonable endodontic obturation material.

## Introduction

The purpose of root canal treatment is to eliminate intracanal microorganisms and to fill the cleaned and shaped root canal spaces. An ideal impervious seal of the root canal space, is a primary goal for endodontic obturation materials that prevents coronal, apical, and lateral penetration of fluids and microorganism [1, 2].

Gutta-percha (GP) is the most widely used obturation material and considered as root filling material of choice. Interestingly, some investigators reported some levels of toxicity for GP [3, 4]. Because of the practical impossibility of thermal sterilization [5], nanosilver coating was suggested for adding antibacterial effects to GP. Significant antibacterial and antifungal properties against *S. Aureus*, *E. Coli*, *E. Faecalis* and *C. Albicans *as well as limited antimicrobial effects on *P. Aeruginosa *were reported for Nanosilver coated GP (NS-GP). However, conventional GP had no antimicrobial or a minimal effect at best [6].

Anti-bacterial and anti-fungal properties of endodontic obturation core materials is of critical importance because of a variety of factors including impossibility of thorough cleaning and shaping of root canal system and possible bacterial influx into an obturated root canal space following to coronal leakage or procedural endodontic mishaps [7]. Unfortunately, acquisition of the aforementioned characteristics may compromise biocompatibility of endodontic filling materials far beyond the tissue tolerance limits [8-10] that may prohibit or postpone periapical tissue healing if obturating materials pass through the apical foramen [11]. Until now, there has been no data on the biocompatibility of NS-GP. So, to make more accurate decision on the material use, the aim of this study was to compare biocompatibility of NS-GP with conventional GP in subcutaneous tissues in rat model.

## Materials and Methods

**Figure 1:**
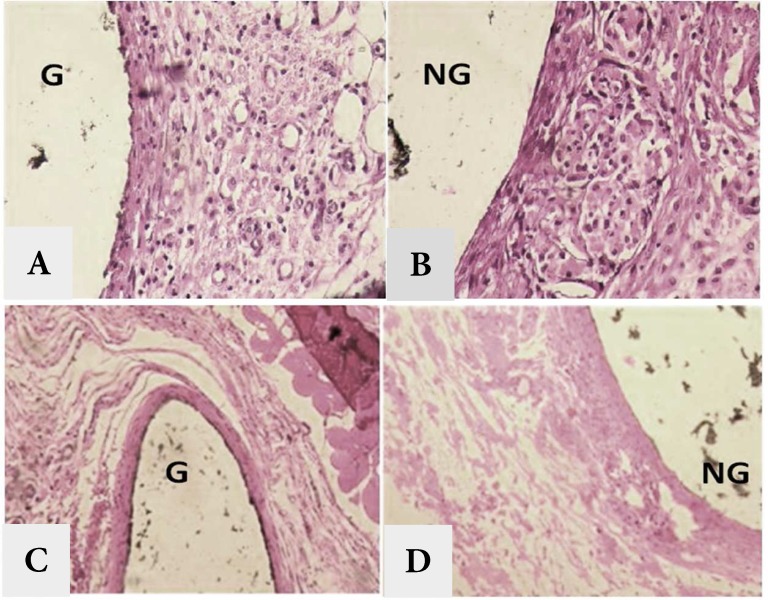
A and B) ×400 view of subcutaneous tissues after 7 days; C and D) ×200 view of subcutaneous tissues after 30 days. (G: gutta-percha; NG: nanosilver coated gutta-percha)

The protocol of this study was approved by the Research Ethics Committee of Iranian Center for Endodontic Research, Shahid Beheshti Dental School, Tehran, Iran (Ethic code:125). Twenty male Wistar rats with approximate weight of 200±20 g were divided into two groups of 10 each. General anesthesia was obtained by using intramuscular injections of 0.5 g/100 mL of ketamine HCl (Ketaject; Phoenix Pharmaceutical, Inc, St Joseph, MO, USA) and Xylazine (AnaSed; Lloyd Laboratories Inc, Shenandoah, IA, USA). All procedures were performed under strict aseptic conditions. Dorsal region of each rat was shaved and disinfected with 0.12% chlorhexidine gluconate. In the surgical site, left and right skin incisions of equal sizes were made and subcutaneous pockets prepared by blunt dissection for receiving GP (Aria Dent, Tehran, Iran) or NS-GP (Iran Polymer and Petrochemical Institute, Tehran, Iran). The test materials were sterilized with ethylene oxide and were equal in size (30/0.02) and length (10 mm). Two animals were assigned as controls which had incisions made without placement of the test materials. For approximation of incisional wound edges 4-0 nylon sutures were used. The animals were sacrificed after 7 and 30 days with an anesthetic overdose. Implants and surrounding tissues were carefully excised and transferred to 10% neutral buffered formalin (pH=7.4) for a 48-h fixation process. The solution was replenished after 24 h. After fixation, tissue samples were embedded in paraffin. Longitudinal serial sections of 5 to 7 µm were prepared and stained with Hematoxylin and Eosin (Figure 1). Light microscopic histologic evaluation (Leica DME, Leica Microsystems Inc., Buffalo, New York, USA) of the tissue sections was blindly accomplished by two independent and calibrated oral and maxillofacial pathologists. Inflammatory response was scored based on type and count of inflammatory cells (none=0, mild=1, moderate=2 and severe=3) [12]. Moreover, necrosis, abscess and fibrous connective tissue formation were recorded. Regarding the presence of different variables, four statistical analyses were used, namely Mann-Whitney U, Wilcoxon Signed Ranks, Fisher Exact, and McNemar’s tests. 

## Results

Table1 and 2 show inflammatory reaction in experimental groups. After 7 days mild and moderate inflammation was observed in conventional GP and NS-GP, respectively. Mann-Whitney U test showed significant difference between two groups (*P=*0.001). While dominant type of inflammatory cells for conventional GP was lymphocyte and plasma cell, neutrophils and lymphocytes were prominent in NS-GP which indicated the acute inflammatory reaction to the latter (Figure 1). According to the Fisher Exact test, there was a statistically significant difference between inflammatory cell types between two materials (*P*=0.001).

After 30 days, no inflammatory reaction was detected in conventional GP. However, a mild inflammation was reported for NS-GP. Mann-Whitney U test showed a statistically significant difference between two groups (*P*=0.001). No inflammatory cell was detected in conventional GP. Lymphocyte and plasma cell comprised the prominent cell types in immune cell infiltration of NS-GP (Figure 1). Fisher Exact test reported a significant difference in inflammatory cell types between two groups (*P*=0.001).

**Table1:** Inflammatory reaction after 7 and 30 days in different experimental groups

	**GP**	**NS-GP**	***P-*** **value**
**Day 7 (degree/percent)**	Mild (100)	Moderate (100)	0.001
**Day 30 (degree/percent)**	None (100)	Mild (100)	0.001
***P *** **value (degree/percent)**	0.002	0.002	

**Table 2:** Inflammatory infiltration after 7 and 30 days in different experimental groups

	**GP**	**NS-GP**	***P-*** **value**
**Day 7 **	Lymphocyte, Plasma cell	Lymphocyte, Neutrophil	0.001
**Day 30 **	-	Lymphocyte, Plasma cell	0.001
***P *** **value **	0.002	0.002	

Conventional GP showed mild inflammation (including lymphocytes and plasma cells as key cellular components of inflammatory reaction) on the 7th day. However, after 30 days no inflammation (no presence of inflammatory cells) was reported for this group. Wilcoxon Signed Ranks test showed a statistically significant difference in inflammation rate between 7th day and 30th day (*P*=0.002). According to the McNemar test, a significant difference was shown in type of inflammatory cells between two periods (*P*=0.002).

Inflammatory reaction towards NS-GP was moderate (including neutrophils and lymphocytes) on the 7th day which turned into a mild type (mainly made up of lymphocytes and plasma cells) after 30 days. Wilcoxon Signed Ranks test showed a significant difference in inflammation rate between two trial periods (*P*=0.002). McNemar test showed that there was a statistically significant difference in the inflammatory cell types between two periods (*P*=0.002).

After 7 days, formation of granulation tissue and blood vessels was evident in both groups. However, the amount of this granulation tissue formation was greater in NS-GP. After 30 days, replacement of this granulation tissue by fibrous-collagenous connective tissue, a sign of healing, was reported in both groups. 

## Discussion

Root canal filling materials are dispensable part of contemporary endodontics. Obturation materials should meet toxicity and biocompatibility standards which are significant biologic aspects of clinical use. Although antimicrobial effects of root canal filling materials may have some merits of microorganism elimination, their unintended cytotoxicity can also be harmful to the host cellular populations. Limited antibacterial spectrum and concerns about antibiotic resistant bacterial strains impose limitations on intracanal use of antibiotics. Antiseptics such as silver are wide spectrum and because of nonspecific targeting at cellular processes, bacterial resistance is not a matter of importance [13, 14].

To date, GP is the most popular core root canal filling material. Physical, chemical and biologic properties have been reported for nanosilver particles (typically smaller than 100 nm and contain 20-15000 silver atoms). Ancient physicians believed that silver powder had a healing effect and anti-disease benefits [15]. One of its modern applications is to synthesize composites for use as antibacterial water filters [16]. There is an increasing use of silver as an efficacious antibacterial and antifungal agent in wound care products and medical devices [17-20].

Modern advances in nanotechnology eliminated the possibility of irreversible pigmentation of the skin and eyes through long-term exposure to silver [21]. High antimicrobial effect of nanosilver particles is the main reason for their application to reduce microbial colonization in dental implants, vascular devices and urinary catheters [22-25]. Activity of human mesenchymal cells and blood clotting effect were increased by nano-particles that may result in better wound healing [26, 27]. Today's silver base dressings are available in the forms of creams, foams, hydrogels, hydrocolloids, polymeric layers and meshes [14]. Nanotechnology advancements offer the opportunity to increase surface area interactions; the more the available surface area of nanosilver particles, the more antibacterial effects are anticipated. Bactericidal effects have been demonstrated for nanosilver particles of 5-50 nm [28].

Greulich *et al. *[26], evaluated biocompatibility of nanosilver particles and reaction of human mesenchymal cells (hMSCs) to these particles. Activation of hMSCs occurred at lower concentrations of 2.5 μg and toxic effects appeared at higher concentrations of 5 μg. They concluded that ascending concentration of nanosilver particles reduced proliferation and chemotaxis of hMSCs.

Shantiaee *et al. *[29] reported that the toxicity of NS-GP in L929 fibroblasts decreased over time. After 7 days, there was no statistically significant difference between NS-GP and conventional GP. Implantation of endodontic filling materials into the subcutaneous connective tissue in rats is considered a valid secondary screening test for biocompatibility [30-32]. The present study was conducted on rats which mimic human tissue reactions to the test materials more accurate than MTT assays.

In a study by Bodrumlu *et al. *[12], moderate and mild subcutaneous inflammation were reported for GP after 30 and 60 days, respectively. They reported an inverse relationship between inflammation rate and thickness of fibrous capsule. In the present study no inflammation was evident after 30 days. This difference may be because of different test materials and method of sterilization. In this study, inflammatory reaction decreased over time that supports the results of previous studies [12, 33, 34]. Eventually, mild inflammation was reported only for NS-GP. In addition, fibrous-collagenous tissue formed around inflammation area after 7 days which had thickened in both groups throughout the study. 

## Conclusion

After 7 days, inflammation rate and type of inflammatory cells differed between two groups. Nanosilver coated GP showed higher level of inflammation than conventional GP. After 30 days, conventional GP had no inflammation. However, nanosilver coated GP showed a mild inflammatory reaction. Both test materials were surrounded by fibrous connective tissue which represented a sign of healing. Nano-silver coated GP showed biocompatibility in subcutaneous connective tissues of rat.
